# Red-Crowned Crane (*Grus japonensis*) Reproduction Was Improved by Inhibiting Mycotoxins with Montmorillonite in Feed

**DOI:** 10.3390/toxins12030191

**Published:** 2020-03-18

**Authors:** Dawei Liu, Chao Gu, Changhu Lu, Qinghua Wu, Kamil Kuca, Wenda Wu

**Affiliations:** 1 Nanjing Forest Police College, Nanjing 210023, China; dwliu@nfpc.edu.cn; 2 College of Biology and the Environment, Nanjing Forestry University, Nanjing 210037, China; luchanghu@njfu.com.cn; 3 Key Laboratory of State Forest and Grassland Administration Wildlife Evidence Technology, Nanjing 210023, China; 4 MOE Joint International Research Laboratory of Animal Health and Food Safety, College of Veterinary Medicine, Nanjing Agricultural University, Nanjing 210095, China; guchao0990@163.com; 5 College of Life Science, Yangtze University, Jingzhou 434025, China; wqh212@hotmail.com; 6 Department of Chemistry, Faculty of Science, University of Hradec Kralove, 50003 Hradec Kralove, Czech Republic

**Keywords:** red-crowned crane, reproduction, mycotoxin, montmorillonite

## Abstract

The red-crowned crane (*Grus japonensis*) is a vulnerable bird species. Mycotoxins are toxic substances produced by filamentous fungi and are considered as naturally unavoidable contaminants in animal feed. Our recent survey indicated that feeds designed for captive red-crowned cranes were contaminated with mycotoxins. This study was conducted to investigate the protective effects of the mycotoxin binder montmorillonite on the reproductive behavior, sex hormone levels, and egg quality of red-crowned cranes. Twelve pairs of *G. japonensis* were divided into four groups, and each group was fed one of the following: a selected diet (with extra low levels of mycotoxins), a regular diet, a selected diet with 0.5% montmorillonite added, or a regular diet with 0.5% montmorillonite added. Consumption of the regular diet decreased courtship and mating behaviors, testosterone concentration, egg weight, and shell thickness. However, feed supplementation with montmorillonite increased the courtship, mating behaviors and testosterone concentration during the pre-breeding period, as well as egg weight and shell thickness. These findings suggest that the addition of dietary montmorillonite is effective for controlling mycotoxins in the feed, resulting in improvements in reproductive behaviors, testosterone concentrations, and some egg quality parameters of the red-crowned crane.

## 1. Introduction

Contamination of animal feeds and feed ingredients with mycotoxins is a global concern [1]. Animals that eat foods contaminated with high levels of toxins may exhibit obvious symptoms of poisoning. However, according to previous studies, high doses of mycotoxins in animal feeds are uncommon [2,3]. Instead, repeated exposure to low levels of mycotoxins is a significant concern, as the animal does not exhibit obvious clinical symptoms but the health level and reproductive ability continue to decline. In China, the concentration of allowable aflatoxins in animal feed is 6-14 μg/kg, which is lower than that in many other countries and is within a recognized safety range. However, Jones et al. [4] conducted toxin surveys and assessments of several poultry farms and found that toxins within the safety range had adverse effects on animal health. Moreover, multiple types of mycotoxins can be present in a bag of feed; even if the concentration of each toxin in the feed is very low, the interaction between toxins can adversely affect animal health [5]. Therefore, safe levels and limits do not protect animals, as mycotoxins do not currently have a safe lower limit. Higher levels of toxins are a greater health risk to animals [6].

Few foods are completely free of mycotoxins. Thus, determining whether to feed animals with foods that are entirely free of mycotoxins on a daily basis is not possible. Currently, as there is no established “safe dose” of mycotoxins in wild animal feeds, wildlife breeding institutions must refer to the national limit set for livestock and poultry, which generally have significantly shorter life cycles than most captive wild and exotic animals. However, the long-term intake of low levels of toxins can affect their health and reproductive capacity, even if no significant clinical symptoms are apparent [7].

Most studies have focused on the effects of mycotoxins on animal health [8,9,10,11,12]. However, some studies have revealed that toxins can damage the reproductive performance of animals. For example, T-2 toxin (T-2) can decrease the thickness of a hen’s eggshell [13]. Aflatoxins (AFs) can cause laying hens to stop laying eggs [14], and zearalenone (ZEN) elicits an estrogenic effect that disrupts animal reproductive function and cause a series of reproductive disorders in animals [15]. Ebrahem et al. [16] suggested that low levels of mycotoxins may not affect the reproductive performance of birds; instead, toxins are transmitted to the eggs from the mother, causing the embryo to die during development. 

In order to control mycotoxin contamination, many detoxifying methods and technologies including chemical, biological, and physical approaches have been developed [17]. For instance, some clays are added to feed to prevent the absorption of mycotoxins from the gastrointestinal tract and their distribution to blood or target organs [18]. Because of inefficiency and environmental problems induced by biochemical production, the practical applications of chemical and biological approaches have been limited to control mycotoxin contamination [19]. Contrarily, physical approach draws great attention due to the outstanding detoxification performance, low expense, and biocompatibility advantages [20]. A commonly used physical binder is montmorillonite. This acidic aluminosilicate clay has two tetrahedral sheets of silica sandwiching a central octahedral sheet of alumina [21]. The formula of this clay is (Na,Ca)0,3(Al,Mg)2Si4O10(OH)2·n(H2O). It is thought that the silanol, aluminol, and hydroxyl groups on the clay edges and permanent charged sites on the basal surfaces act as adsorption sites [22]. Experimental data have shown that montmorillonite can be effective in counteracting the toxic effects of polar mycotoxin [23,24].

Red-crowned crane (*Grus japonensis*) is a globally threatened species [25]. At present, approximately 2750 migratory individuals exist in the wild, and breed in the northeast of China and the far east of Russia, with a large portion overwintering in Yancheng, China [26,27]. This bird is among the most popular wildlife species and has gained overwhelming support from the Chinese people during the national bird selection process. To prevent the extinction of this threatened species, a re-introduction program involving “artificial feeding, captive breeding and subsequent release” may be effective [28]. In China, great efforts have been made to maintain and breed the animal in many zoos, wetlands and reserves [29]. For example, in unnatural distribution regions, the natural environment of the bird’s breeding ground has been simulated to promote reproduction [30].

Under natural conditions, the reproduction of these birds is seasonal, and illumination is an important environmental factor that regulates both reproductive behavior and hormone levels via the endocrine system of the birds [31,32]. Increasing the illumination time increased the testosterone levels of male birds during the non-breeding season, and continuous long-term illumination resulted in increased semen production [33]. Illumination during the simulated breeding period allowed non-breeding females to enter the breeding season [34]. For a wide variety of cranes, increasing the illumination time may stimulate reproduction [35]. The environment in Yancheng—the red-crowned crane’s wintering ground—was supplemented with artificial light to increase egg production by captive cranes, which gradually led to increases in the population. 

According to a survey, in some areas of China, including Yancheng, captive red-crowned crane feeds were all contaminated by mycotoxins. Of all the analyzed feed samples, the incidence of aflatoxin B_1_ (AFB_1_), deoxynivalenol (DON), ZEN, T-2, and ochratoxin A (OTA) was 43.54%, 90.32%, 93.55%, 69.35%, 74.20%, respectively; the mean level detected was 7.62, 871.49, 616.34, 65.62 and 14.50 µg/kg, respectively, and the co-occurrence of at least two mycotoxins was also observed in almost all samples [36]. The results suggested a risk for the precious crane. In this study, we examined the protective effects of montmorillonite on the reproductive behavior, sex hormone levels, fertilization rate, hatching rate and health rate of these birds during the breeding season to minimize the impact of pollutants in the diet on their reproduction.

## 2. Results

### 2.1. Dietary Mycotoxin Concentration

In this study, the cranes were divided into four treatment groups, which were selected diet; selected diet + 0.5% montmorillonite; regular diet; regular diet + 0.5% montmorillonite. All dietary mycotoxin concentrations of AFB_1_, DON, ZEN, T-2, and OTA are shown in Table 1. Apart from OTA, the levels of the other four toxins in the selected diet and selected diet + 0.5% montmorillonite groups were lower than those in the regular diet and regular diet + 0.5% montmorillonite groups. In all groups, OTA was under the limit of quantification.

### 2.2. Reproductive Behavior

Throughout the trial period, all cranes were in good health with no diseases and no deaths. At different stages of the breeding season, the reproductive behavior of the cranes (Table 2 and Table 3) was significantly different. No nesting behavior was observed in the pre-breeding stage, and few mating, courtship, or nesting behaviors were noted in the post-breeding stage. Therefore, these behaviors are not listed in the tables.

In the pre-breeding stage, no significant differences (*p* > 0.05) were found between the courtship and mating behaviors of the red-crowned cranes in the selected diet + 0.5% montmorillonite and the selected diet groups. Similarly, the regular diet + 0.5% montmorillonite and the selected diet groups did not significantly differ with respect to mating behavior (*p* > 0.05). In contrast, the two behaviors was significantly decreased (*p* < 0.05) in the regular diet group (51.5%, 54.8%), compared with the selected diet group (Table 2). In the mid-breeding stage, there were no significant differences (*p* > 0.05) in the courtship and mating behaviors between the selected diet and selected diet + 0.5% montmorillonite groups, whereas both behaviors in the regular diet were significantly lower than those in the selected diet group (*p* < 0.05). The courtship behavior in the regular diet + 0.5% montmorillonite group was not significantly different from that in the selected diet and regular diet groups (*p* > 0.05). The mating behavior in the regular diet + 0.5% montmorillonite group was significantly (*p* < 0.05) increased in the regular diet group (126.5%), with no significant difference compared to the selected diet group (*p* > 0.05). No significant differences (*p* > 0.05) were found in the nesting and incubation behaviors of the treatment groups in the mid-breeding stage, and no significant differences were observed in the incubation behaviors of the treatment groups at the end of the breeding period (Table 3). This shows that consumption of a regular diet containing higher levels of mycotoxins decreased courtship and mating behaviors. Montmorillonite supplementation in the diet was beneficial for increasing the reproductive behaviors.

### 2.3. Sex Hormone Levels

**Figure 1:**
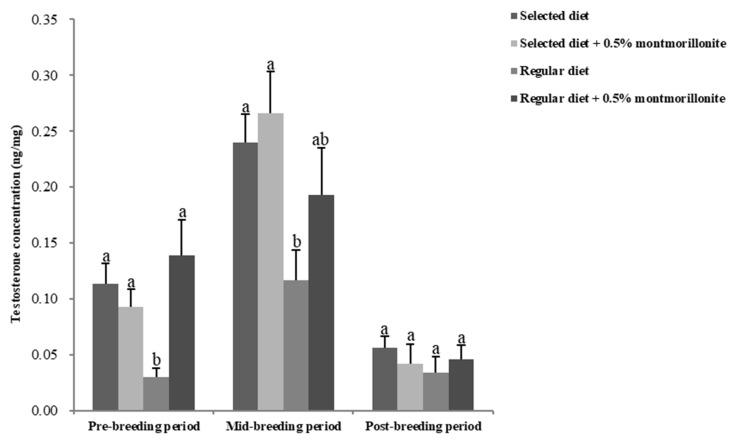
Testosterone levels determined in feces in male red-crowned cranes. Data are mean ± SEM (*n* = 3/group). Bars without the same letter are significantly different (*p* < 0.05).

During the entire breeding period, the testosterone levels in the male red-crowned crane feces in the four treatment groups varied, with lower levels during the pre-breeding period, higher levels during the mid-breeding period, and lower levels during the post-breeding periods. In pre- and mid-breeding phases, the testosterone levels of the male cranes in the regular diet group were significantly decreased (*p* < 0.05) during the pre- (73.6%) and mid-breeding (51.5%) phases as compared to the selected diet group. During the post-breeding period, compared with the regular diet group, only numerical decrease in the hormone level was observed in the regular diet group (*p* > 0.05) (Figure 1). The addition of montmorillonite to the regular diets restored the concentration of this hormone to the level in the selected diet group during the pre- and mid-breeding periods.

**Figure 2:**
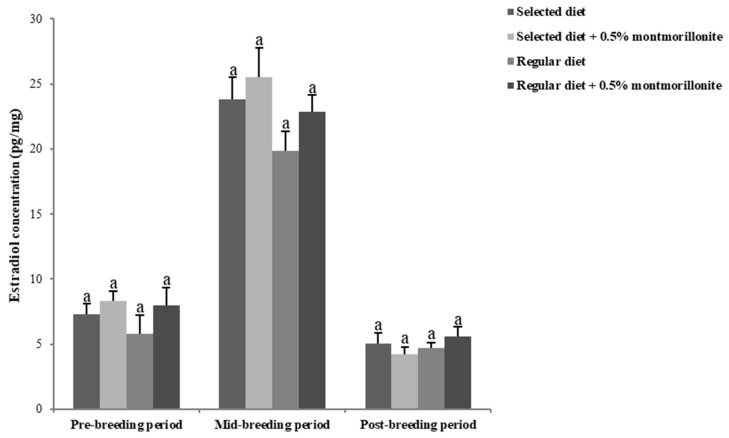
Estradiol levels determined in feces in female red-crowned cranes during the breeding period. Data are mean ± SEM (*n* = 3/group). Same letters above the bar indicate that they are not significantly different (*p* > 0.05).

**Figure 3:**
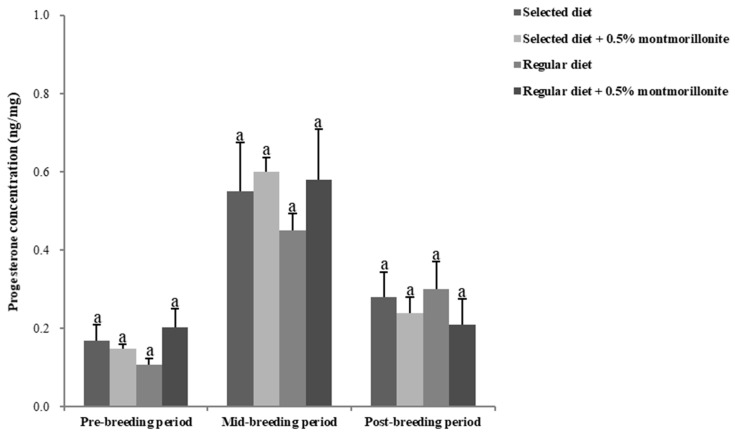
Progesterone determined in feces levels in female red-crowned cranes during the breeding period. Data are mean ± SEM (*n* = 3/group). Same letters above the bar indicate that they are not are significantly different (*p* > 0.05).

As with the changes in male sex hormones, the levels of estradiol and progesterone in the feces of female red-crowned cranes also exhibited lower levels during the pre-breeding, higher levels during the mid-breeding period, and lower levels during the post-breeding periods in the four treatment groups. There were no significant differences (*p* > 0.05) in the levels of these hormones among the treatment groups in female cranes during the breeding period (Figure 2 and Figure 3). 

### 2.4. Egg Quality 

No significant difference (*p* > 0.05) was found in the number of eggs laid by cranes among the treatment groups. The effects of montmorillonite on the quality of crane eggs are shown in Table 4. No significant difference (*p* > 0.05) in the egg weight and eggshell thickness was observed between the selected diet and selected diet + 0.5% montmorillonite groups. However, both of these indices in the regular diet group were significantly lower (*p* < 0.05) than those in the other groups. The regular diet + 0.5% montmorillonite group did not differ in these indices from selected diet group (*p* > 0.05).

These results indicate that addition of montmorillonite to the selected diet did not affect the weight and eggshell thickness of crane eggs. Mycotoxins in a regular diet reduced egg quality and eggshell thickness, and the addition of montmorillonite significantly (*p* < 0.05) improved the effects of toxins on both these indicators.

There were no significant differences in the egg shape index, fertilization rate, hatching rate, and health rate among the treatment groups (*p* > 0.05).

## 3. Discussion

According to the National Feed Safety Standards of China, the highest allowance of AFB_1_, DON, ZEN, T-2, and OTA in poultry feed are 20, 5000, 500, 1000, and 100 μg/kg, respectively [37,38,39]. Compared with these standards, the toxin levels in both the selected and regular diet did not exceed the limits. In this study, the selected diet was chosen after rigorous testing to ensure that the toxin concentration was less than the limit of quantification. To maintain the long life cycle of wildlife, their diets should contain low or no mycotoxins. However, it is difficult to prepare such diets during the actual operation process, and captive cranes are typically fed diets that meet national feed hygiene standards but include several mycotoxins [36]. Thus, it is essential to avoid toxin poisoning. This study is the first to describe the protective effects of montmorillonite on the reproduction of the red-crowned crane. However, the results in this study show that the montmorillonite could only improve the reproduction of red-crowned crane, not eliminate the influence caused by mycotoxins. The possible reason might be that montmorillonite had a low effectiveness on DON and T-2, which still affected the reproduction of red-crowned crane. In future studies, new methods including modified montmorillonite and biodegradation should be developed for the decontamination of T-2 and DON [40,41].

Sex hormones function to promote the maturation of animal sexual organs and maintain secondary sexual characteristics. Typically, the ovary of the female bird is the primary secretory organ of sex hormones, and secretes estrogen (estradiol and estrone) and progesterone. The testes of male animals are the primary secretory organs of androgens and secrete testosterone. Therefore, we monitored these hormones to gain a better understanding of animal gonad function [42]. Many studies have shown that mycotoxins adversely affected the gonads of animals [14,43,44]. In this study, the courtship and mating behaviors of the red-crowned crane in the regular diet group were significantly reduced in the pre- and post-breeding periods. Concomitantly, we observed a significant decrease in testosterone levels in the males in the regular diet group during these two periods. Because reproductive behaviors such as courtship and mating are significantly associated with sex hormone levels [45,46,47], these results suggest that contaminants in the regular diets affect the gonads of the male animals [43], leading to decreased sex hormone secretion [48,49]. Moreover, contaminants in the diet may have affected other hormones and their functions, such as non-steroidal hormones [50]. Under the combined action of such hormones, the reproductive behavior of these animals was somewhat abnormal. However, because of the adsorption of harmful substances by montmorillonite, these reproductive behaviors and hormone levels in the regular diet + 0.5% montmorillonite group returned to levels similar to those in the selected diet group.

The thickness of the eggshell and concentration of the egg contents are important indicators of egg quality [51]. Eggshell thickness decreased by varying degrees during incubation. Normally, eggshell thickness is measured before the eggs are hatched. However, this measurement may be destructive to the chicks, which is not a suitable option for rare wild animals such as red-crowned cranes. Thus, in this study, we measured the thickness of all eggshells after hatching (for viable eggs). The measurement of egg content concentration is also destructive. Therefore, we did not conduct this test.

In this study, it was found that mycotoxins negatively impacted on the egg weight and eggshell thickness, which are similar to previous findings [52,53,54]. The reason for loss of egg weight is the presence of toxins, which affects the normal metabolism of the liver, thereby inhibiting both protein synthesis and lipogenesis [55,56]. Moreover, toxin exposure leads to decreased feed intake and liver damage [57]. Eggshells of birds are composed of calcareous and calcium carbonate particles secreted by the uterine muscle wall. This membrane is wrapped around the eggshell membrane and protects the embryo, which ensures normal development. In our study, the decreased eggshell thickness in the cranes may be related to the toxin-induced deterioration of calcium and phosphorus absorption, vitamin D3 metabolic disorders, and decreased parathyroid hormone levels [58].

A previous study showed that egg weight and eggshell thickness are important factors affecting the hatching rate [51]. Although mycotoxins significantly reduced egg weight and eggshell thickness in this study, the hatching rate was not significantly different among treatment groups. The egg weight and eggshell thickness tests are more important in commercial poultry production which uses machines for large-scale hatching rather than in rare wild animals that are hatched on a smaller scale. Furthermore, poultry hatching is carried out using an incubator at a relatively constant temperature, humidity, and time. These hatching parameters are set for most species of eggs and do not take abnormal eggs into account. Therefore, if the eggs are too heavy or too light, the eggshells become too thick or too thin, respectively, affecting the heat dissipation and evaporation of the embryos. In turn, this affects embryonic development and eventually leads to a decrease in the hatching rate. During the hatching process of the red-crowned crane eggs in the current study, most factors such as heat and humidity were equal, and the main variable was the cooling temperature; this variation may have caused a difference in the degree of development and heat dissipation rate in each egg. However, overall, the cooling rate positively impacted the hatching rate because of differences in egg weight and eggshell thickness and maximized the hatching rate of fertilized eggs.

The rate of healthy chicks formed is another important indicator of egg quality. Studies have shown that mycotoxins in parental foods can be transmitted to the embryo, which then hinder protein synthesis, and cause abnormal body development and malformation [59]. Moreover, these toxins affect the maturation and differentiation of embryonic immune cells, inhibit antibody production, reduce the immunity of offspring, and cause death during development or shortly after birth [60]. In this study, there were no developmentally malformed cranes. Although all experimental groups contained weak chicks, the difference in the rate of healthy chicks born between groups was not significant. This revealed that although contaminants in the food may be transmitted to the body of the embryo, this low dose did not adversely affect normal chick development or its immune function. Thus, although montmorillonite adsorbed mycotoxins in the regular diets, this was not reflected in the hatching of healthy chicks. In the present study, the weak chicks in each test group exhibited a large abdomen and poor yolk absorption. This may have been caused by an excessively period of egg cooling or insufficient accumulated temperature, resulting in a late embryo [61].

## 4. Conclusions

In summary, our data demonstrate that the addition of montmorillonite in regular diets provides protection for the reproductive behaviors, testosterone concentration, and some egg quality parameters of the red-crowned crane; this is the first study to report these findings. From a public health perspective, these findings can be applied to improve the health of red-crowned crane using montmorillonite. 

## 5. Materials and Methods 

### 5.1. Study Area

The study was conducted in the Yancheng Biosphere Reserve, which was established for red-crowned cranes conservation and is recognized as an international biosphere reserve by the United Nations Educational, Scientific, and Cultural Organization.

### 5.2. Chemicals, Animals and Diet

Montmorillonite (Montmorillonite K-10, Cas: 1318-93-0) was obtained from Sigma-Aldrich (St. 182 Louis, MO, USA). As the red-crowned crane is a rare wild animal, the number of captive animals is relatively small. Therefore, in this study, we selected 12 pairs of red-crowned cranes that were 4-6 years old; these cranes were paired successfully and randomly divided into 4 treatment groups, with each group comprising 3 pairs of cranes. Each pair was fed in separate cages separated by black shade nets. All experiments and protocols used in this study were approved by the Yancheng Biosphere Reserve Institutional Animal Care and Use Committee (Identification code: YCDDHKY 001; date of approval: December 22, 2014). The temperature and relative humidity of the test cages were consistent with the environmental conditions.

To stimulate reproduction in cranes, natural light was combined with artificial light. A 60 w (22.5 lx) incandescent lamp was installed at the top of each cage. Illumination was provided via the lamp every day from 4:00 to 7:00 and from 17:00 to 19:00, and the animals were subject to natural light for the remaining time. Before the formal test, the animals in each group were pre-fed for 7 days, after which the formal test was conducted for a total of 122 days.

To increase the hatching rate, we allowed the parent crane to incubate for 7 days and then sent the eggs to the hatchery for artificial hatching.

The diets for each treatment group were composed of: selected diet, prepared by screening several batches of raw materials to ensure that the concentration of mycotoxins in the diets was with extra low level of mycotoxins; selected diet supplemented with 0.5% montmorillonite (selected diet + 0.5% montmorillonite); regular diet; regular diet supplemented with 0.5% montmorillonite (regular diet + 0.5% montmorillonite). 

Both the selected and regular diet consisted of corn, barley, soybean meal, wheat bran, fish meal, salt, shell powder, calcium hydrogen phosphate, and trace element premix. The nutritional level was prepared by referring to the nutrient standard of the breeding season recommended by Ellis et al. [32]. The ingredients and nutrient composition of the diet are shown in Table 5. The diet form was a pellet with a particle size of approximately 10 mm. After the feed was prepared, it was dried and placed in a plastic drum with a tight seal. All essential nutrients in the diets met the standard nutritional specifications for red-crowned cranes. 

### 5.3. Determination of Mycotoxin Concentration in Diets

The dietary concentrations of AFB_1_, DON, ZEN, T-2, and OTA were analyzed at MOE Joint International Research Laboratory of Animal Health and Food Safety, College of Veterinary Medicine, Nanjing Agricultural University, Nanjing. The concentrations of the five toxins in the diet were tested by HPLC (Shimadzu, Shimadzu Corp., Tokyo, Japan) method as described by Liu et al. [62].

### 5.4. Behavioral Observations

For the convenience of analysis, and according to the laws regarding laying cranes in the breeding center of Yancheng Biosphere Reserve, the breeding period for red-crowned cranes was divided into the pre-breeding period (March), mid-breeding period (April, May, early June), and post-breeding period (mid-June and late June). During the observation period, we divided the behaviors of cranes into reproductive and non-reproductive behaviors. The reproductive behaviors included courtship, mating, nesting, incubation (spawning or hatching). The non-reproductive behaviors included foraging, feeding, swallowing, drinking, walking, flying, jumping, running, bathing, shaking feathers, spreading wings, flapping wings, spreading unilateral legs, standing, and walking. 

To observe the animals without disturbing their natural behaviors, an instantaneous scanning method was employed to observe and record the crane behaviors in each cage at 5-min intervals. From March 1, 2015, to June 30, 2015, animal behaviors were observed every other day for 58 days. The observation period lasted 12 h from 6:00 to 18:00 on each observation day. A total of 8304 scans were performed, including 2160 pre-breeding, 4848 mid-breeding, and 1296 post-breeding period scans. To reduce the impact of extreme weather on our results, observations were halted during heavy rain, strong winds, and other inclement weather conditions.

### 5.5. Determination of Sex Hormones in the Feces

Fecal collections were performed while observing the behavior of the red-crowned cranes on Saturdays or Sundays during the study. All fecal collections were performed at 8:00–10:00 (Beijing time) to reduce the effect of different time intervals on the daily rhythm of individual hormone secretions. 

Specific method: Following defecation of the target animal, the feces were collected quickly without disturbing the animal. The gravel, weeds, and feathers were removed, and the samples were stored at −20°C until analysis.

The frozen fecal samples were thawed at room temperature, and then the samples of the pre-fertility, mid-breeding, and post-fertility samples for each individual were thoroughly mixed. For testosterone (T) and progesterone (P) extraction: 0.8 g of fecal sample was mixed with 7 mL of methanol and 0.8 mL of distilled water, and shaken for 3 min. Next, 4 mL of petroleum ether was added and shaken for 1 min, followed by centrifugation at 1,500 rpm for 10 min. We removed 5 mL of the methanol layer, which was stored at −20°C until analysis. For estradiol (E2) extraction, 0.8 g of fecal sample was mixed with 6.5 mL of dichloromethane and 3.5 mL of NaOH, shaken for 5 min, and centrifuged at 1500 rpm for 10 min. Next, 5 mL of the methylene chloride layer was removed and washed twice with 1.5 mL of distilled water. We evaporated 3 mL of the methylene chloride layer in a 45 °C water bath, and reconstituted the sample with 0.5 mL of phosphate buffer prior to analysis. T, P, and E2 were measured by radioimmunoassay (RIA) using an RIA kit purchased from the Beijing Huaying Biotechnology Research Institute. The hormone measuring instrument was an r-911 automatic release counter (China University of Science and Technology Industrial Corporation, Hefei, China).

### 5.6. Measurement and Calculation of Egg Index

The weight of the egg was weighed using an electronic balance prior to hatching.

To measure eggshell thickness, all eggshells were collected after the chicks were out of the shell. The shells were immersed in hot water at 60–80°C for 20 min to remove the shell membrane. After washing and drying, the thickness of the sharp, middle, and blunt ends of the eggshell was measured with a caliper, and the average value of the three parts was the value used as the eggshell thickness. 

The egg shape index, fertilization rate, hatching rate, and healthy chick rate were determined using the following formulas: Egg shape index = long diameter/short diameter; Fertilization rate = the number of fertilized eggs/the number of hatching eggs × 100%; Hatching rate = the number of colts hatched/ number of fertilized eggs × 100%; Healthy chick rate = number of healthy colts hatched/ number of chicks hatched × 100%.

### 5.7. Incubation of Eggs

Before hatching, a hatching machine (EI DF 4200 incubator, Qingdao, China) and hatching room were thoroughly fumigated and disinfected. Following egg removal from the nests, the eggs were wrapped in sterile cotton wool, placed in egg storage boxes, and quickly sent to the hatchery. Bumps were avoided during transportation. At the hatchery, the eggs were placed into the incubator after proper disinfection for artificial hatching of the eggs.

The incubation room temperature was maintained at ~25°C, and relative humidity was maintained at ~60%. The incubator temperature was 37.6–37.7 °C from the time of hatching to shelling; the relative humidity was 45%. The egg was automatically turned over once every 2 h by an incubator at an angle of 90°. Immediately after breaking of the shell, the eggs were moved to the hatching basket in the hatcher. The temperature was set to 37.2–37.4 °C, and the relative humidity was 50–55%.

The eggs were cooled once every 3 h from 6:00 to 24:00, whereas at other times, the eggs were cooled once every 4–6 h to simulate the parent leaving the nest for food. Until hatching, cooling times for the eggs were 3–5 min within 10 days, 6–10 min at 11–20 days, and 10–15 min after 21 days.

### 5.8. Statistics

The data were analyzed using SPSS 16.0 statistical software (SPSS, Inc., Chicago, IL, USA). The percent-type variables (fertilization rate, hatchability, and rate of health) were inversely sinusoidal and transformed prior to analysis. All indicators were tested for normality using Shapiro–Wilk test. Following testing, the differences between treatments were analyzed by one-way analysis of variance and least significant difference tests were compared multiple times. The data were tested for homogeneity of variance prior to analysis of variance. The test data were expressed as the mean ± standard error (mean ± SE). The differences were considered significantly different at *p* < 0.05.

## Figures and Tables

**Table 1:** Mycotoxin concentrations in experimental diets.

Dietary Treatment	Mycotoxin (μg/kg)				
	AFB_1_	DON	ZEN	T-2	OTA
Selected diet	ND ^a^	16.4 ± 8.3	9.4 ± 3.8	<LOQ ^b^	<LOQ
Selected diet + 0.5% montmorillonite	ND	18.1 ± 7.8	11.7 ± 5.2	ND	<LOQ
Regular diet	7.3 ± 2.5	776.7 ± 95.3	479.9 ± 38.7	51.6 ± 13.6	<LOQ
Regular diet + 0.5% montmorillonite	6.7 ± 3.1	875.2 ± 85.9	486.6 ± 43.4	62.7 ± 17.8	<LOQ

^a^ ND = not detected; ^b^ LOQ = limit of quantification. Data are mean ± SEM (*n* = 3/group).

**Table 2:** Reproductive behaviors in red-crowned cranes in the pre-breeding period.

Dietary Treatment	Courtship Times	Mating Times	Nesting Times
Selected diet	60.3 ± 6.7 ^a^	24.0 ± 4.7 ^a^	45.0 ± 6.7 ^a^
Selected diet + 0.5% montmorillonite	72.0 ± 4.4 ^a^	23.3 ± 2.6 ^a^	40.3 ± 6.2 ^a^
Regular diet	36.7 ± 4.9 ^b^	11.7 ± 2.0 ^b^	25.7 ± 5.2 ^a^
Regular diet + 0.5% montmorillonite	58.3 ± 9.0 ^a^	26.5 ± 4.5 ^a^	38.0 ± 7.2 ^a^

^a,b^ Values with different superscripts in the same row are significantly different (*P* < 0.05). Data are mean ± SEM (*n* = 3/group).

**Table 3:** Reproductive behaviors in red-crowned cranes in mid- and post-breeding periods.

Dietary Treatment	Mid-Breeding Period				Post-Breeding Period
	Courtship	Mating	Nesting	Laying and/or Hatching	Laying and/or Hatching
Selected diet	26.0 ± 2.7 ^a^	12.3 ± 2.5^a^	4.3 ± 3.8 ^a^	822.3 ± 31.9 ^a^	57.0 ± 4.6 ^a^
Selected diet + 0.5% montmorillonite	23.3 ± 5.8 ^ab^	8.0 ± 2.3 ^ab^	5.7 ± 2.6 ^a^	851.7 ± 75.7 ^a^	72.0 ± 7.0 ^a^
Regular diet	11.3 ± 2.0 ^b^	3.7 ± 0.9 ^b^	6.3 ± 2.0 ^a^	789.0 ± 28.7 ^a^	65.3 ± 4.1^a^
Regular diet + 0.5% montmorillonite	24.0 ± 4.7 ^ab^	10.7 ± 4.5 ^a^	2.3 ± 1.5 ^a^	774.7 ± 67.4 ^a^	60.7 ± 5.6 ^a^

^a,b^ Values with different superscripts in the same row are significantly different (*p* < 0.05). Data are mean ± SEM (*n* = 3/group).

**Table 4:** Breeding and egg characteristics of red-crowned cranes.

Dietary Treatment	Number of Eggs (n)	Egg Weight (g)	Shell Thickness (mm)	Egg Shape Index	Fertilization (%)	Hatching Rate (%)	Healthy Chick Rate (%)
Selected diet	2.7 ± 0.7 ^a^	264.1 ± 3.1 ^a^	0.6 ± 0.01 ^a^	1.6 ± 0.0 ^a^	83.3 ± 16.7 ^a^	72.3 ± 14.7 ^a^	91.7 ± 8.3 ^a^
Selected diet + 0.5% montmorillonite	4.7 ± 0.9 ^a^	256.5 ± 2.0 ^a^	0.5 ± 0.01 ^ab^	1.5 ± 0.0 ^a^	91.7 ± 8.3 ^a^	100.0 ± 0.0 ^a^	89.0 ± 11.0 ^a^
Regular diet	3.7 ± 1.3 ^a^	234.7 ± 5.2 ^b^	0.5 ± 0.02 ^b^	1.6 ± 0.0 ^a^	75.0 ± 14.4 ^a^	100.0 ± 0.0 ^a^	72.3 ± 14.7 ^a^
Regular diet + 0.5% montmorillonite	4.3 ± 1.7 ^a^	258.3 ± 2.6 ^a^	0.6 ± 0.02 ^a^	1.6 ± 0.0 ^a^	91.7 ± 8.3 ^a^	83.3 ± 16.7 ^a^	91.7 ± 8.3 ^a^

^a,b^ Values with different superscripts in the same row are significantly different (*p* < 0.05). Data are mean ± SEM (*n* = 3/group).

**Table 5:** Ingredients and nutrient composition of red-crowned crane diet during breeding season.

Ingredient	Percentage	Nutrition Index	Value
Corn	41.2	Metabolic (Mcal/kg)	2.73
Barely	12.5	Crude protein (%)	21.2
Soybean meal	23	Calcium (%)	2.57
Wheat bran	10	Phosphor (%)	0.96
Fish meal	5		
Sodium chloride	0.3		
Premix *	1		
Shell power	3		
Calcium phosphate	4		

* Premix contained the following per kilogram of diet: limestone, 3500 mg; copper, 220 mg; iron, 2500 mg; cobalt, 30 mg; zinc, 2000 mg; manganese, 3000 mg; iodine, 60 mg; vitamin A, 200,000 IU; vitamin B2, 180 mg; vitamin B6, 60 mg; vitamin B12, 0.3 mg; vitamin K3, 80 mg; vitamin E, 200 IU; pantothenic acid, 200 mg; niacin, 300 mg; biotin, 2 mg; folic acid, 25 mg; Choline chloride, 20 mg; L-lysine HCl, 33 g; DL-methionine, 15 g.
